# Mechanism of Filament Nucleation and Branch Stability Revealed by the Structure of the Arp2/3 Complex at Actin Branch Junctions

**DOI:** 10.1371/journal.pbio.0030383

**Published:** 2005-11-08

**Authors:** Coumaran Egile, Isabelle Rouiller, Xiao-Ping Xu, Niels Volkmann, Rong Li, Dorit Hanein

**Affiliations:** **1**Department of Cell Biology, Harvard Medical School, Boston, Massachusetts, United States of America,; **2**Cell Adhesion Program, Burnham Institute for Medical Research, La Jolla, California, United States of America,; **3**The Stowers Institute for Medical Research, Kansas City, Missouri, United States of America; UCSF Medical CenterUnited States of America

## Abstract

Actin branch junctions are conserved cytoskeletal elements critical for the generation of protrusive force during actin polymerization-driven cellular motility. Assembly of actin branch junctions requires the Arp2/3 complex, upon activation, to initiate a new actin (daughter) filament branch from the side of an existing (mother) filament, leading to the formation of a dendritic actin network with the fast growing (barbed) ends facing the direction of movement. Using genetic labeling and electron microscopy, we have determined the structural organization of actin branch junctions assembled in vitro with 1-nm precision. We show here that the activators of the Arp2/3 complex, except cortactin, dissociate after branch formation. The Arp2/3 complex associates with the mother filament through a comprehensive network of interactions, with the long axis of the complex aligned nearly perpendicular to the mother filament. The actin-related proteins, Arp2 and Arp3, are positioned with their barbed ends facing the direction of daughter filament growth. This subunit map brings direct structural insights into the mechanism of assembly and mechanical stability of actin branch junctions.

## Abstract 2

Genetic labeling and electron microscopy were used to examine actin branch junctions assembled in vitro. The subunit map obtained offers insights into the assembly of these conserved cytoskeletal elements.

## Introduction

The Arp2/3 complex is a key cytoskeletal regulator of actin polymerization [1]. The complex promotes the assembly of dendritic actin networks that drive cell locomotion, phagocytosis, and intracellular motility of lipid vesicles, organelles, and invasive pathogens [2]. Conserved among eukaryotes, this seven-subunit, 220-kDa complex consists of two actin-related proteins, Arp2 and Arp3, and five additional subunits named ARPC1 through ARPC5. The isolated complex has a low nucleation activity, but upon binding to nucleation promoting factors (NPFs), ATP, and preexisting (mother) actin filaments, the Arp2/3 complex promotes the formation of a branched actin structure where the complex itself is situated at the branch junction [3,4].

Despite intensive study, the mechanistic details of the branch junction formation are still poorly understood, partly because of the lack of high-resolution information about the structure of the activated conformation of the complex at the branch junction. Two speculative models have been proposed for subunit organization of the Arp2/3 complex at these branch junctions. Information used for the modeling included sequence conservation among species, available biochemical and structural information, and, most important, the hypothesis that Arp2 and Arp3 assume an actin filament dimer-like configuration that templates the initiation of the daughter filament in the barbed end direction [5,6]. Another conceptually different model, derived primarily from kinetic analysis, suggested that the Arp2/3 complex induces branching and elongation at the barbed end of growing filaments with Arp2 and Arp3 being incorporated in two different actin filaments [7]. However, no direct structural data were available to support any of the proposed nucleation models.

We provide here the first structural analysis to our knowledge of the Arp2/3 subunit organization at the branch junction at molecular resolution using genetic labeling, electron microscopy, and computational analysis. We show that various NPFs, except cortactin, dissociate from the complex after branch formation and that all of the Arp2/3 subunits are in a position to contact the mother filament. In contrast to the previous attempts to model the orientation of Arp2/3 within the actin branch, we have not assumed that Arp2 and Arp3 are orientated toward the daughter filament. Thus, our unbiased subunit localization provides direct evidence that Arp2 and Arp3 are positioned with their barbed ends facing the direction of daughter filament growth.

## Results/Discussion

A direct observation of the complex within the branch junction at molecular resolution is required to better understand the mechanism of branched actin nucleation by the Arp2/3 complex. The general strategy that we have taken to achieve this goal was to assemble actin branches in vitro using a complex with one of the subunits carrying a label that can be detected by electron microscopy. The location of the label (and the corresponding subunit) in the image plane can be determined by difference mapping between the two-dimensional (2D) projection maps of branches assembled with labeled and unlabeled complexes.

### The WASp-Family NPFs, but Not Cortactin, Detach from the Arp2/3 Complex after Branch Formation

**Figure 1:**
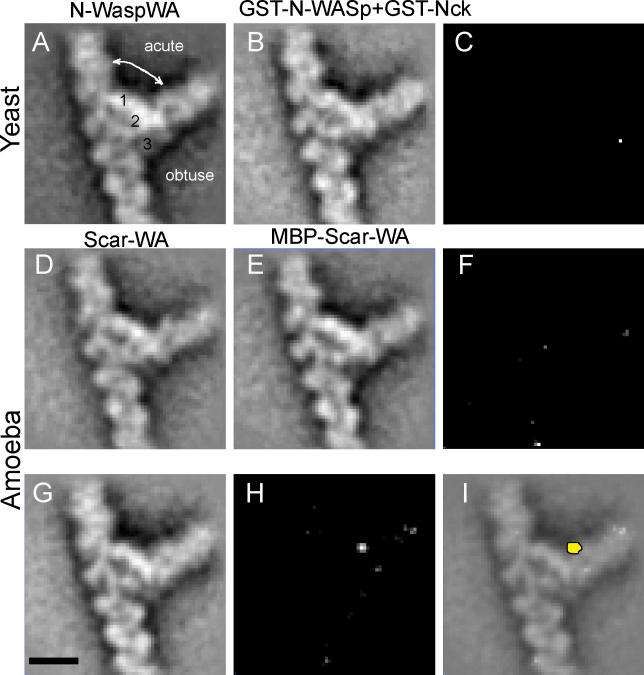
Visualization of NPFs in the Actin Branch Junction by Difference Mapping (A-F) WASp protein dissociate from the actin branch. 2D average projection obtained (A) using the unlabeled yeast Arp2/3 complex and N-WASp WA, and (B) using the full-length GST-N-WASp complexed with its activator GST-Nck. (C) Difference map between (A) and (B). 2D average projection obtained using (D) the amoeba Arp2/3 complex and Scar WA, and (E) MBP-Scar1WA. (F) Difference map between (D) and (E). (G-I) Cortactin is present in the actin branch. (G) 2D average projection obtained using GST-cortactin and the amoeba Arp2/3 complex. (H) Difference map between (G) and (D). (I) The peak in the difference map shown in yellow superimposed with the projection map. Bar = 10 nm.

We first employed this strategy to compare branch junctions formed in the presence of different NPFs of increasing molecular weight. Difference mapping would allow detection of the additional density contributed by the larger NPF, allowing localization of the NPF in the branch junction. We assembled actin branches with *Saccharomyces cerevisiae* Arp2/3 complex or *Acanthamoeba* complex in the presence of WASp-family NPFs of various sizes that contained the Arp2/3-activating region WA (WASp homology 2 and acidic motifs) (Figure S1). These were N-WASp WA (~12 kDa), glutathione-*S*-transferase (GST)–N-WASp WA (~40 kDa), WAVE1/Scar WA (~12 kDa), maltose binding protein (MBP)–tagged WAVE1/Scar WA (~55 kDa), full-length N-WASp (GST-N-WASp bound to its activator GST-Nck and forming a complex of ~153 kDa), and a non-WASp activator, cortactin. Projection images of the branches were boxed, aligned, and averaged to yield two-dimensional (2D) projection maps of the branch junction structure with a resolution of approximately 2.2 nm (Figure 1). The resolution was estimated based on the Fourier ring correlation criteria with a cut-off value of 0.5. Interestingly, no statistically significant differences (at 99.5% confidence level, *p* = 0.005) were observed between the density maps of branches assembled with various WA proteins (12 to 153 kDa) (Figure 1A-1F), whereas a clearly visible, statistically significant difference was observed with GST-cortactin (90 kDa) (Figure 1G-1I). The ability to detect the activator was verified by difference maps using free activated complexes selected from the same electron microscope grids from which the branches were selected (X.-P. X., D. H., and N. V., unpublished data).

The additional density attributed to cortactin was located on the obtuse side of the branch next to the main bridge of density on the daughter filament side (Figure 1I). Cortactin enhances the persistence of lamellipodia protrusion during cell motility [8] and probably promotes this effect by stabilizing Arp2/3 branches induced by WAVE2/Scar2 [9]. Thus, the localization of cortactin at the branch junction provides a mechanism for stabilizing either the Arp-mother or the Arp-daughter interaction. We favor a stabilization of the Arp-mother interaction, as this would explain the nucleation promoting effect of cortactin on the Arp2/3 complex. However, the relatively weak signal observed with GST-cortactin construct precludes determination of the molecular nature of cortactin interactions with the mother or the daughter filament. Our localization positioned the construct density at a site consistent with the idea that cortactin might bind to the Arp3 subunit. The absence of WASp-family NPFs at the branch junction, as revealed by the difference maps, is consistent with the observation that N-WASp/WASp-coated beads undergo motility by cycles of binding, activation, and release of the Arp2/3 complex [10,11].

### Localization of Arp2, Arp3, Arc40/ARPC1, and Arc18/ARPC3 Subunits at Actin Branch Junction

**Figure 2:**
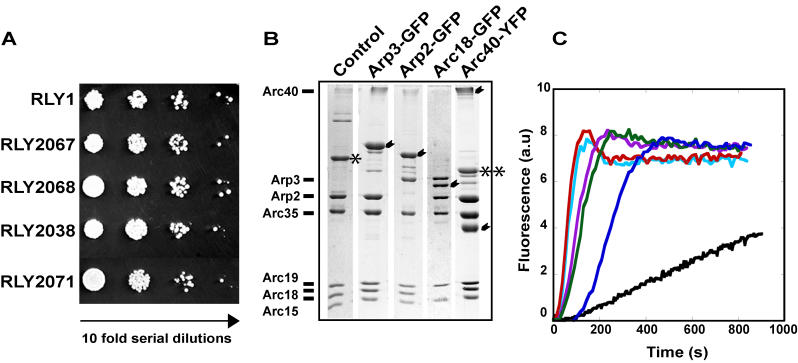
Characterization of GFP- or YFP-Labeled Yeast Arp2/3 Complexes (A) Serial dilutions of yeast strain cultures expressing Arp3-GFP, Arp2-GFP, Arc40-YFP, and Arc18-GFP Arp2/3-labeled complexes, as well as the unlabeled control complex, were grown on YPD plates at 30 °C. (B) Purified yeast Arp2/3 complexes visualized by SDS-PAGE and Coomassie blue staining; unlabeled (control), and GFP- or YFP-labeled Arp3, Arp2, Arc40, or Arc18 complexes. The labeled subunits are marked by arrowheads. The Arc40 subunit in the labeled Arc40/ARPC1-YFP complex ran as 30-kDa and high-molecular-weight species (previously confirmed by immunoblotting and peptide sequencing), owing to an unusual electrophoretic mobility [16]. The Arp3 subunit of the unlabeled complex is denoted by an asterisk, and the Arp3 subunit of labeled Arc40/ARPC1-YFP complex is denoted by two asterisks. (C) Pyrenyl-actin polymerization kinetics obtained with actin alone (black), control complex (light blue), Arp3-GFP complex (red), Arp2-GFP complex (purple), Arc40/ARPC1-YFP complex (green), and Arc18/ARPC3-GFP complex (dark blue).

**Figure 3:**
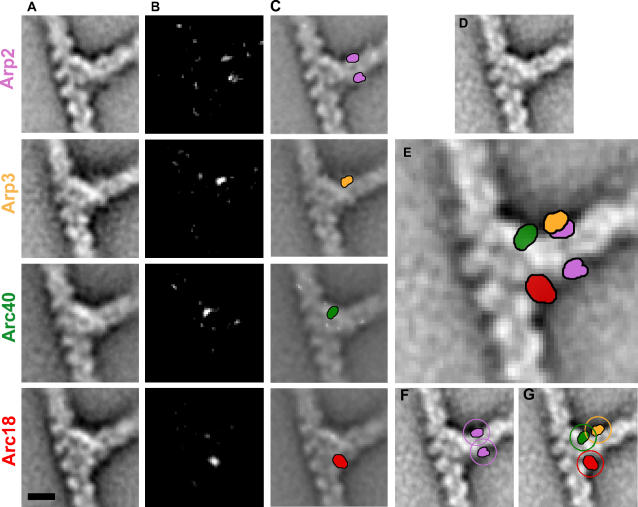
Localization of the Labels Attached to Arp2, Arp3, Arc40/ARPC1, and Arc18/ARPC3 at the Actin Branch Junction Color codes used: Arp2 (pink), Arp3 (orange), Arc40/ARPC1 (green), and Arc18/ARPC3 (red). (A) 2D average projection maps of the branches obtained with Arp2-GFP (row 1), Arp3-GFP (row 2), Arc40/ARPC1-YFP (row 3), and Arc18/ARPC3-GFP (row 4). (B) Difference maps calculated between maps obtained with labeled and unlabeled complexes. (C) Difference maps superimposed with the projection maps. The position of the difference peaks was cross-validated (see Materials and Methods). (D) The average projection map obtained with the unlabeled complex. (E) The main difference peaks are superimposed with the unlabeled projection map. (F and G) Circles of 3.9-nm radius centered on the difference peaks indicate the possible locations of the C-termini of each labeled subunit. The GFP/YFP label was attached to the C-terminus of the relevant subunit with an eight-amino-acid flexible linker that in fully extended conformation can reach a length of up to approximately 3.2 nm. The distance of the N-terminus of GFP or YFP from the center of mass of its beta-barrel (14 × 8 × 8 nm) is approximately 2.5 nm. The centers of the peaks determined from the difference maps probably coincide with the center of mass. Bar = 10 nm.

To locate Arp2/3 complex subunits in the branch junction by difference mapping, we took a genetic approach to introduce a label to individual subunits of the yeast Arp2/3 complex. Yeast genes encoding Arp2, Arp3, Arc40/ARPC1, and Arc18/ARPC3 subunits were tagged with green fluorescent protein (GFP) or yellow fluorescent protein (YFP) coding sequence at their genomic loci through homologous recombination. The C-terminus of each labeled subunit was separated from the label by an eight-amino-acid linker. The advantages of this strategy over the more traditional gold-labeling methods are that our strategy allows highly efficient labeling (~100%) of each subunit and convenient assessment of the functionality of the labeled complex. All four GFP/YFP-tagged strains grew normally at room temperature (not shown) and 30 °C compared to the unlabeled (control) strain (Figure 2A). The GFP label also contained a (His)_10_ tag at the C-terminus, allowing purification of the labeled complex by Ni-NTA affinity (Figure 2B). The unlabeled control Arp2/3 complex was also isolated by Ni-NTA affinity. The nucleation activities of these complexes were tested using the pyrene-actin polymerization assay in the presence of GST-N-WASp WA. The labeled complexes exhibited the same level of nucleation activity as the unlabeled complex (Figure 2C). Actin branches were assembled in the presence of the unlabeled complex or each of the labeled Arp2/3 complexes. Projection maps of the branch junction structures at a resolution of approximately 2.2 nm were generated (Figure 3). Difference maps between branches obtained with the labeled complexes and the unlabeled complex were calculated (Figure 3B and 3C). For cross-validation, each dataset was analyzed independently by two different operators using two different image analysis protocols (Figure S2). All difference maps contain peaks in the branch junction that are statistically significant at a confidence level of 99.5% (*p* = 0.005) using Student's *t*-test. The sizes of the peaks are consistent with the presence of an additional protein of the size of a GFP or YFP monomer (~30 kDa).

In the 2D projection of actin branch junctions, the Arp2/3 complex forms three bridges of density between the mother and daughter filament: a strong bridge of density on the side of the acute angle, a weak bridge of density on the side of the obtuse angle, and a medium bridge of density in the middle (see Figure 1A) [3]. The difference maps between the projection densities obtained from the labeled complexes and the control complex showed that the YFP attached to Arc40/ARPC1 was present on the main bridge close to the mother filament, the GFP attached to Arp3 was on the same side but further away from the mother filament, and the GFP attached to Arc18/ARPC3 was located on the weak bridge close to the mother filament. The GFP attached to Arp2 generated two statistically significant peaks in the difference maps of Arp2-GFP: one located on the obtuse angle of the branch, and the other on the acute side. The two peaks correspond to two alternative stable positions of the GFP, because the population of the Arp2-GFP branches can be sorted into two clusters that each show only one peak (Figure S3). Both GFP positions are compatible with the same C-terminus location owing to the length of the flexible linker (Figure 3F).

### Determination of the Orientation of the Arp2/3 Complex at the Branch Junction by Computational Modeling

**Figure 4:**
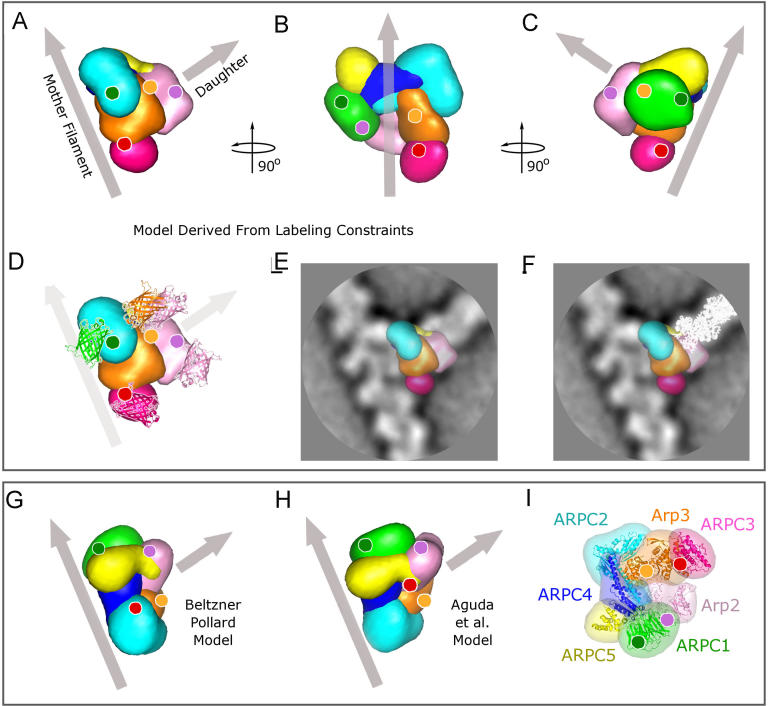
Structure Models of the Arp2/3 Complex at Actin Branch Junction Color codes used: Arp2 (light pink), Arp3 (orange), Arc40/ARPC1 (green), Arc35/ARPC2 (cyan), Arc19/ARPC4 (blue), Arc18/ARPC3 (dark pink), and Arc15/ARPC5 (yellow). Gray arrows indicate the mother and daughter filaments. (A) Orientation of the Arp2/3 complex relative to the mother and daughter filaments as determined using the labeling constraints. (B) Model rotated vertically anticlockwise by 90° from view in (A) and tilted so that the mother filament coincides with the vertical axis. The gray arrow is positioned to pass through the center of the complex. (C) Model rotated vertically by 180° from the view in (A). (D) Label positions and their corresponding C-termini localization. GFP or YFP, shown as ribbon diagram with the same color coding as in Figure 3, were superimposed on the respective difference peak (Figure 3) with their orientation matching the peak shape. (E) Model superimposed on the projection density map (white corresponds to high density). (F) Model and ribbon diagram of a daughter filament (white) as it would grow after small relative rotations of Arp2 and Arp3 (see text) superimposed on the projection density map. (G and H) Model proposed by Beltzner and Pollard [6] (G) and by Aguda et al. [5] (H) shown for comparison. Note that in (G), the daughter filament will be oriented out of the paper plane toward the reader. (I)Arp2/3 crystal structure in the same orientation as originally presented in Robinson et al. [12].

For all difference maps, the peaks correspond to a projection onto the image plane *(XY)* of the respective center-of-mass position of the label. Despite the lack of information on the out-of-plane *(Z)* coordinates, we can use the *XY* coordinates of the centers of mass as efficient constraints for the three-dimensional (3D) orientation of the complex, because the *XY* projection of the C-terminus of each labeled subunit must fall within a distance defined by the length of the covalently attached linker and the topology of the label (Figure 3E-3G). In the branch junction, all of the individual positions must be satisfied simultaneously. For example, the C-terminus of Arp2 needs to be in a position that allows attachment to GFP at both positions detected in the difference maps. This restricts the possible *XY* projection of the Arp2 C-terminus to the small area where the distance to both peaks is below the cut-off distance (i.e., the common area of the two circles in Figure 3F). Addition of constraints for the other subunits further reduces the number of compatible orientations. A global orientation search with the crystal structure of the inactive Arp2/3 complex [12] was carried out to map all orientations that are compatible with the given constraints. The results revealed only a single cluster of orientations that satisfied the label constraints (Figure 4) with an estimated precision of approximately 1 nm. In all permissible orientations, domains I and III of both Arp2 and Arp3, corresponding to the fast growing (barbed) end in an actin filament, are facing away from the mother filament toward the daughter filament**.**

The relative orientations of Arp2 and Arp3 would need to change from that in the inactive structure in order to provide a suitable template for the growth of the daughter filament. The exact nature of these changes are unknown, but the amplitudes of the changes detected so far [13] are small enough to argue against massive subunit rearrangements such as dissociation of Arp2 and rebinding to Arp3 in a long-pitch filament conformation. The preservation of overall topology of the complex is supported by the fact that all of the constraints obtained in this study for the positions of the labels can be satisfied without the need to introduce changes in the relative orientation of the subunits in the inactive complex. Consistent with a conformational change upon activation, the relative orientation of both Arp2 and Arp3 would need to be altered in our model to provide an exact match of the daughter filament with the direction of its projection density. This conformation could be achieved by an approximately 15° rotation of Arp3 around its short axis and an approximately 15° rotation of Arp2 around an axis parallel to its short axis passing through domain I of Arp3, accompanied by a slight adjustment of the overall complex orientation (<5°). Even though this rearrangement corresponds to a substantial conformational change in the Arp2/3 complex, these rotations would be fully compatible with the constraints imposed by the labels, leading to displacements of the labeled C-termini projections by less than the estimated precision (<1 nm). The resulting daughter filament would not only grow parallel to the *XY* plane and coincide with the direction of the daughter filament in the projection maps but also fit the shape of the projection density remarkably well (Figure 4E and 4F). Domains II and IV of Arp3 (orange in Figure 4) are well positioned to make direct contact with the mother filament. The other Arp2/3 subunits are also in a position to contact the mother filament, with Arp2 (pink) being the furthest away from the mother filament (Figure 4A-4D). The fact that Arp3 is close to the mother filament was not apparent from the projection images alone [3] and could not be inferred from other available data. The data from subunit labeling, in conjunction with the crystal structure of the isolated complex, allowed a much more detailed and accurate assignment of the densities than previously possible and indicate that the previous assignment was one unit off (i.e., the previous Arp3 position corresponds to Arp2 and Arp2 to the first actin monomer in the daughter filament).

### Conclusions

The data presented here support the model that Arp2 and Arp3 adopt an actin short-pitch dimer-like configuration that templates the initiation of the daughter filament in the barbed end direction. The data are incompatible with the proposed incorporation of Arp2 and Arp3 into two different actin filaments at the branch junction [7]. The two available hypothetical structural models of the branch junction [5,6] (illustrated in Figure 4G and 4H) relied on the assumption that the barbed ends of Arp2 and Arp3 face the daughter filament to orient the complex within the branch junction. In contrast, the labeling-based model presented here did not use this assumption as a constraint, and therefore our results lend unbiased evidence to the proposed mechanism where Arp2 and Arp3 serve as a template dimer for the barbed-end-directed growth of the daughter filament. Additionally, our localization data are incompatible with the positions of the C-termini of the subunits proposed in both these previous models (compare Figure 4A with 4G and 4H). These models suggested that the longest axis of the complex, comprising ARPC1, −5, −4, and −2 (Arc40, −15, −19, and −35 in the yeast Arp2/3 complex), contacts the side of the mother filament (Figure 4G and 4H). Our model deviates from these models by an anticlockwise rotation of approximately 100° around the axis of the daughter filament, resulting in an alignment of the longest axis almost perpendicular rather than parallel to the mother filament (Figure 4A). This geometry could allow comprehensive interactions between the axis formed by ARPC2/4 (with possible contribution from ARPC5) and a groove of the mother filament, with Arp3 and ARPC3 on one side and ARPC1 on the other side to provide stabilizing interactions that would prevent the complex from rocking horizontally as well as vertically against the mother filament.

In summary, our model provides the structural basis for the mechanical stability of branch junction that is important for effective force generation upon filament elongation at the barbed ends. It is fully consistent with the available biochemical data and the growth direction of the daughter filament and directly supports the template-dimer model of Arp2/3-mediated actin nucleation. The subunit map established in this analysis thus provides a new structural framework for further understanding the spatial and temporal control of branch nucleation and turnover in the generation of an advancing dendritic network that drives protrusive cellular movement.

## Materials and Methods

### Plasmids, genetic manipulations, and yeast strains

Yeast strains expressing C-terminal GFP- or YFP-labeled Arp2/3 complex subunits were generated by homologous recombination by the integration of a cassette containing a linker (GDGAGLIN), the yEGFP (or yECitrine) coding sequence, and a polyhistidine (His)_10_ tag at the 3′ end of each open reading frame. The cassette was generated using pCE36, a derivative of pKT128 [14]. Strains used in this study are listed in Table 1.

**Table 1:**
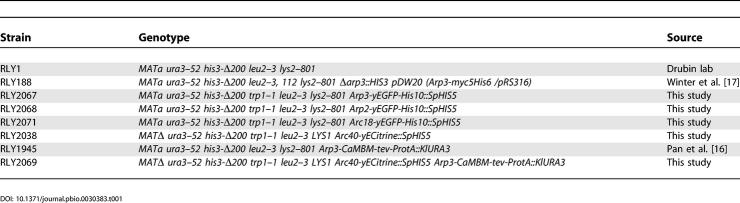
Yeast Strains Used in This Study

### Proteins

Actin was purified from rabbit muscle and isolated as Ca^2+^-ATP-G-actin in G buffer (5 mM Tris-Cl [pH 7.8], 0.1 mM CaCl_2_, 0.2 mM ATP, and 1 mM DTT) according to Pardee and Spudich [15] and pyrenyl labeled. The yeast Arc40/ARPC1-YFP Arp2/3 complex was isolated from a strain expressing an Arp3-CaMBM-tev-ProtA subunit (RLY1945) as previously described [16]. The unlabeled control complex (Arp3MH, which has a [Myc]_5_His_6_ tag on Arp3 [16]) as well as the Arp3-, Arp2-, and Arc18/ARPC3-GFP-His_10_-labeled complexes were isolated as follows. Yeast cells were grown to mid log phase in YPD medium (OD_600_ 2−4) washed in U buffer (50 mM HEPES [pH 7.5], 100 mM KCl, 3 mM MgCl_2_, and 1 mM EGTA) and stored at −80 °C until use. A 50- to 100-g cell pellet was resuspended in five volumes of cold U buffer supplemented with 0.5% Triton X-100, 0.2 mM ATP, 1 mM DTT, and protease inhibitor mix (0.5 μg/ml antipain, leupeptin, pepstatin A, chymostatin, and aprotinin, and 1 mM PMSF) and passed through a microfludizer (Microfluidics, Newton, Massachusetts, United States) until 70% lysis was obtained. The cell extracts were cleared by centrifugation at 100,000 × g for 1 h and filtered through a 0.45-μm filter. A 60% ammonium sulfate precipitation of cell extracts was performed, and the pellet was dialyzed into NaP buffer (100 mM phosphate [pH 7.8], 100 mM KCl, and 20 mM imidazole). This fraction was cleared by centrifugation and incubated with Ni-NTA agarose beads (Qiagen, Valencia, California, United States). Beads were washed with NaP buffer, NaP buffer plus 0.5 M KCl, and NaP buffer plus 0.5% Triton X-100, and the complex was eluted with 250 mM imidazole. The complex was further purified through a HiTrapS column (Amersham Biosciences, Little Chalfont, United Kingdom) in 50 mM MES (pH 6.5), 25 mM NaCl; a UnoQ1 column (Bio-Rad, Hercules, California, United States) in U buffer; and a Superose 12 gel filtration column (Amersham Biosciences) in U buffer on a BioLogic chromatography system (Bio-Rad). *Acanthamoeba* Arp2/3 complex was purified by poly(L)-proline [18] and gel filtration chromatography as described [19]. Purified complexes were immediately used to assemble actin branches or stored in U buffer supplemented with 0.2 M sucrose, flash frozen in liquid nitrogen, and stored at −80 °C. Bovine GST-N-WASp WA, bovine GST-N-WASp, murine GST-cortactin, and murine GST-Nck were purified as previously described [20,21]. MBP-Scar1 WA was generated by fusing Scar1 S495–C559 to MBP followed by a C-terminal His_6_ (E. Kim and D. H., unpublished data).

### Pyrene-actin polymerization assays

Pyrene-actin polymerization assays were performed at Harvard Medical School and repeated at the Burnham Institute. Typically, G-actin was clarified at 436,000 × g for 30 min. Reactions were performed by mixing 2 μM Mg^2+^-ATP-G-actin (10% pyrene labeled) with Arp2/3 complex and the appropriate NPF, and actin polymerization was initiated in 1× KMEI buffer (50 mM KCl, 2 mM MgCl_2_, 1 mM EGTA, 0.2 mM DTT, 0.1 mM ATP, 0.02% azide, and 2 mM imidazole [pH 7.0]). Polymerization was followed using a fluorescence spectrophotometer (Cary Eclipse Varian at Harvard Medical School and an MOS-250 spectrofluorometer [BioLogic, Claix, France] equipped with BioKine 32 software at the Burnham Institute), using 365 nm as the excitation wavelength and 407 nm as the emission wavelength. All of the GFP-labeled complexes were used at 50 to 200 nM concentration with 100 to 200 nM GST-N-WASp WA. When the reaction reached the plateau, 2 μM phalloidin was added to stabilize the branches, and the reaction was diluted as required in F buffer (5 mM Tris-Cl [pH 7.8], 50 mM KCl, 1 mM MgCl_2_, 0.1 mM CaCl_2_, 0.2 mM ATP, and 1 mM DTT). The slight reduction in the polymerization rate with Arc18/ARPC3-GFP complex was due to frozen storage of the complex. This reduction was not observed with fresh complex (data not shown), which was used to obtain the actin branches studied using EM. For the NPF detection experiments, we used the following conditions. Yeast actin branches were assembled with 2 μM actin with 25–50 nM yeast Arp2/3 complex activated by either 250 nM N-WASp WA, 250 nM GST-N-WASp WA, or 125 nM GST-N-WASp/250 nM GST-Nck. Amoeba actin branches were assembled with 2 μM actin with 100 nM amoeba Arp2/3 complex activated by either 200 nM Scar WA, 200 nM MBP-Scar WA, or 200 nM GST-cortactin. No actin polymerization was observed when 2 μM G-actin is incubated with 25–50 nM yeast or 100 nM amoeba complexes (data not shown). Activation of the amoeba Arp2/3 complex with GST-cortactin was significantly weaker than the activation observed with Scar WA or MBP-Scar WA.

### Electron microscopy

Freshly purified Arp2/3 complexes were used to assemble actin filament branches, which were applied to glow-discharged EM carbon-coated grids and stained with 2% uranyl acetate. Images were recorded under low-dose conditions at a magnification of 42,000 and at a defocus of 1.8 μm using a Tecnai 12 G2 microscope (FEI, Hillsboro, Oregon, United States) equipped with a Lab6 filament at 120 kV and a 1,024 × 1,024 MSC 600HP (model 794; Gatan, Pleasanton, California, United States). The pixel size was 0.57 nm. Branches were selected and boxed using EMAN [22]. Image analysis was performed independently by two different experimentalists (I. R. and X.-P. X.), using two different image analysis packages: Spider [23] and EMAN [22]. Results were compared only at the end of the analysis.

### Image processing and cross-validation

For alignment using Spider, selected branches were aligned with a reference-based alignment procedure using standard alignment protocols implemented in Spider [23]. The initial reference was a well-stained branch chosen from the dataset. After alignment, branches were inspected visually, outliers (branches that obviously were not aligned) were discarded, and aligned branches were averaged. This new average was used for another round of alignment. This process was repeated until no more changes were observed (typically three or four times). For several datasets (Arp2-GFP, full-length N-WASp, and cortactin), three different initial references (two different branches and the average obtained with the control) were used. Comparison of the different final averages for individual datasets showed that they were practically identical (and the difference map between the average and the control maps showed the same difference peaks), i.e., the final average was not biased by the choice of the initial reference. The other datasets were aligned to one or two initial references and the results were cross-validated with the results from EMAN (see below). Of 109 branches, 103 were included in the unlabeled yeast Arp2/3complex with GST-N-WASp WA averaging, 169 (of 170) in the unlabeled yeast Arp2/3 with N-WASp WA averaging, 111 (of 121) in the unlabeled yeast Arp2/3 complex with GST-N-WASp and GST-Nck averaging, 204 (of 211) in the Arc40/ARPC1-YFP averaging, 173 (of 188) in the Arp2-GFP averaging, 158 (of 163) in the Arp3-GFP averaging, 162 (of 165) in the Arc18/ARPC3-GFP averaging, 205 (of 211) in the amoeba Arp2/3 complex with Scar WA averaging, 135 (of 147) in the amoeba Arp2/3 complex with MBP-Scar WA averaging, and 250 (of 284) in the amoeba Arp2/3 complex with GST-cortactin averaging.

For alignment using EMAN, for the dataset of the unlabeled yeast Arp2/3 branches in the presence of N-WASp WA and amoeba Arp2/3 complex in the presence of Scar WA, initial references with good quality (straight and with high contrast) were picked from the respective dataset. Projection maps were generated using the correlation-based iterative alignment algorithm and outlier screening implemented in EMAN [22]. To further reduce model bias, the procedure was repeated for nine different references each. The final projection maps were generated by aligning and averaging the respective nine maps. For all other datasets, the final projection map of either the amoeba complex in the presence of Scar WA (for amoeba-based samples) or yeast complex in the presence of N-WASp WA (for yeast-based samples) was used as the initial reference. The individual branches used for the analysis (final projection maps versus the total boxed branches) are for Arp2-GFP (84/98), Arp3-GFP (110/159), Arc18/ARPC3-GFP (109/154), Arc40/ARPC1-YFP (134/178), unlabeled Arp2/3 complex with N-WASp WA (105/157), unlabeled Arp2/3 complex with GST-N-WASp WA (76/101), unlabeled yeast Arp2/3 complex with full-length GST-N-WASp with GST-Nck (60/82), amoeba Arp2/3 complex with Scar WA (146/200), amoeba Arp2/3 complex with MBP-Scar WA (94/132), and amoeba Arp2/3 complex with GST-cortactin (187/254).

### Averaging and significance testing

The aligned images selected for averaging (separately for the Spider and EMAN sets) were normalized and averaged using routines from CoAn [24]. CoAn was also used to calculate the difference maps and the standardized variance maps that are suitable for input to Student's *t*-test procedures [25]. All tests were performed at a confidence level of 99.5%. All peaks presented were statistically significant and virtually at the same location in the two independent image analyses.

### Fitting of constraints and precision estimate

In order to computationally fit the constraints obtained by labeling, we adapted routines from the CoAn package [24] that were previously used in the context of density fitting and subsequent evaluation of 3D real-space constraints derived from mutagenesis and biochemistry experiments [26]. The routines, which perform a global scan of the orientations, were modified to handle 2D constraints. After applying a rotation to the crystal structure of the inactive Arp2/3 complex, the positions of the four C-termini were projected onto the *XY* plane. Then, a translational least-squares fit between the projected C-termini and the respective constraints (in-plane positions of the labels, one constraint each for Arc40/ARPC1, Arc18/ARPC3, and Arp3 and two for Arp2) was performed. Next, the distances between the C-termini projections and the respective constraints were tested using a predetermined cut-off value. If the distance was below this value, the orientation was kept for further processing. A complete global scan using a 10° increment with this configuration completes within 3 min on an Athlon Opteron dual processor box. An advantage of a global scan versus the more traditional least-squares fitting is that all solutions that satisfy the constraints are mapped and can be used for solution set analysis similar to that used for density-based docking [27].

To determine an estimate for the uncertainty of the orientation in three dimensions, we used the following procedure. The length of the linker and the 3D structures of GFP and YFP determine that the (projected) distance between the respective C-terminus and the difference peak (assumed to represent the center of the GFP/YFP) can be anywhere between 0 and 6 nm. A priori, we do not know which value to choose, but we can use the following argument to find the most appropriate cut-off. The set of 3D orientations that satisfy the constraints at a certain cut-off value can be used to calculate a central orientation (centroid) that minimizes the average root-mean-square deviation to all other members of the solution set. If the cut-off value is too small, the constraints are too tight and the centroid will be biased toward the tightest constraint. If the cut-off value is too large, the centroid will not change, but the solution set will be too large and give unrealistically large estimates of precision. Thus, the most appropriate cut-off distance is the smallest value that still gives the same centroid orientation as larger values. The solution set from this value can be used to get an estimate of the precision for the orientation determination by calculating the average root-mean-square deviation in the set.

Using this procedure with test cut-off values between 1 and 6 nm, we found that the most appropriate cut-off value is 3.9 nm. The centroid orientation (which is the one presented in Figure 4) has an average in-plane distance between the C-termini and the respective peaks of 2.43 nm (Arp3: 2.59 nm; Arp2: 2.98 and 3.48 nm; Arc40/ARPC1: 1.30 nm; Arc18/ARPC3: 1.78 nm; see also Figure 3). The precision of the 3D orientation was estimated from the solution set as 0.99 nm.

### Molecular graphics

In Figure 4, the low-resolution representations of the Arp2/3 complex were generated from the crystal structure [12]. Coordinates for domains I and II of Arp2 are not available owing to disorder in the crystal structure. We substituted these two domains by subdomains 1 and 2 of an actin monomer [28] after least-squares fitting of subdomains 3 and 4 of actin to domains III and IV of Arp2. Representation of atomic models and densities was done using Pymol (http://www.pymol.org).

## Supporting Information

Figure S1 Activity Curves of the Arp2/3 Complexes with Different NPFs Pyrene-actin polymerization curves obtained with 2 μM actin (pink curves) and 2 μM actin (blue curves) and (A) 25 nM yeast Arp2/3 complex, 250 nM N-WASp WA; (B) 50 nM yeast Arp2/3, 125 nM GST-N-WASp, 250 nM GST-Nck; (C) 50 nM yeast Arp2/3, 250 nM GST-N-WASp WA; (D) 100 nM amoeba Arp2/3, 200 nM Scar WA; (E) 100 nM amoeba Arp2/3, 200 nM MBP-Scar WA; and (F) 200 nM amoeba Arp2/3, 200 nM GST-cortactin. No actin polymerization was observed when 2 μM G-actin was incubated with 25 nM yeast or 100 nM amoeba complexes (data not shown). The time scale of each experiment varied because these were performed with different actin preparations on different days. Activation of the amoeba Arp2/3 complex with GST-cortactin was significantly weaker than the activation observed with Scar WA or MBP-Scar WA.
(7.3 MB TIF).

Figure S2 Cross-Validation of the Position of the Difference Peaks Obtained between Projection Maps Calculated with Labeled and Unlabeled Complexes (A-C) Difference maps obtained using the Spider package (see Materials and Methods). (A) 2D projection maps of the branches obtained with unlabeled and labeled yeast Arp2/3 complexes (rows 1–4). (B) Difference maps calculated between maps obtained with labeled and unlabeled complexes. (C) Difference maps (difference peaks shown in yellow) superposed with the projection maps.
(D-F) Difference maps obtained using the EMAN package. (D) 2D projection maps of the branches. (E) Difference maps between maps obtained with labeled and unlabeled yeast Arp2/3 complexes. (F) Projection maps superimposed with the difference maps (difference peaks shown in blue).
(G) Comparison of the results obtained by Spider and EMAN. The difference peaks of the original experiment, shown in yellow, and the cross-validation experiment, shown in blue, are superimposed with the overlapping area shown in green.
Bar = 10 nm.
(1.8 MB TIF).

Figure S3 Sorting the Population of the Arp2-GFP Branches The Arp2-GFP branches were sorted based on the density values in the two areas (pink peaks shown in Figure 3C and 3F). For each peak, the density within the peak area was measured for every aligned branch image. This resulted in distinct bimodal distributions with one peak at high values (peak present) and another at low values (peak absent). The bimodal character of the distribution indicates that we indeed have a systematic difference; otherwise, a single Gaussian distribution would occur. The averages were then calculated from the subpopulation with high values only.
(A-C) Difference map obtained from all Arp2-GFP branches.
(D–I) 2D average map averages from the data sorted based on higher density in the lower peak (middle row D-F), and in the upper peak (bottom row G-I) are shown.
(J) 2D average map average from the unlabeled yeast Arp2/3 complex.
Columns 2 and 3 are the difference maps between the three Arp2-GFP branch averages and the control average at two different contour levels. The branch numbers used for the averages are 84 in (A), 31 in (D), and 42 in (G) for Arp2-GFP branches and 146 for the control in (J). Bar = 10 nm.
(9.4 MB TIF).

### Accession Numbers

The GenBank (http://www.ncbi.nlm.nih.gov/Genbank) accession numbers for proteins discussed in this paper are *S. cerevisiae* Arc15/ARPC5 (NP_012202), Arc18/APRC3 (NP_013474), Arc19/ARPC4 (NP_012912), Arc35/ARPC2 (NP_014433), Arc40/ARPC1 (NP_009793), Arp2 (NP_010255), and Arp3 (NP_012599), bovine N-WASp (NP_776644), human WAVE1/Scar1 (Q92558), and murine cortactin (Q60598).

## Abbreviations

2D: two-dimensional
3D: three-dimensional
Arp: actin-related protein
GFP: green fluorescent protein
GST: glutathione-*S*-transferase
MPB: maltose binding protein
NPF: nucleation promoting factor
WA: WASp homology 2 and acidic motifs
YFP: yellow fluorescent protein
